# A47 THE GUT MICROBIOTA INFLUENCES COLONIC HEALING AFTER SURGERY IN PATIENTS UNDERGOING BOWEL RESECTION FOR COLORECTAL CANCER

**DOI:** 10.1093/jcag/gwac036.047

**Published:** 2023-03-07

**Authors:** R Hajjar, E Gonzalez, G Fragoso, M Oliero, A A Alaoui, A Calvé, H Vennin Rendos, S Djediai, T Cuisiniere, P Laplante, C Gerkins, A S Ajayi, K Diop, N Taleb, S Thérien, F Schampaert, H Alratrout, F Dagbert, R Loungnarath, H Sebajang, F Schwenter, R Wassef, R Ratelle, E Debroux, J -F Cailhier, B Routy, B Annabi, N Brereton, C Richard, M M Santos

**Affiliations:** 1 Université de Montréal; 2 Human Genetics, McGill University; 3 CRCHUM; 4 UQAM; 5 CHUM, Montreal, Canada; 6 Surgery; 7 Medicine, CHUM; 8 Axe cancer, CRCHUM, Montreal, Canada

## Abstract

**Background:**

The standard treatment of colorectal cancer (CRC) consists of a surgical resection of the colonic segment with the tumor, followed by a reconnection of the remaining bowel ends, or "anastomosis". The anastomosis may fail to heal in up to 20% of patients, which leads to anastomotic leak, a major complication that increases postoperative morbidity and mortality. This complication is unpredictable and its causes remain poorly understood.

**Purpose:**

The objective of this study is to investigate the possible role of the gut microbiome in anastomotic healing after surgery in patients with CRC.

**Method:**

We collected preoperative fecal samples and intraoperative mucosal samples from CRC patients undergoing surgery with anastomosis. The gut microbiota of patients with AL and of others that presented optimal healing after surgery was analyzed and compared using the Anchor 16S pipeline. To assess the role of the patients' microbiota in healing, fecal microbiota transplantation (FMT) was performed in mice using preoperative fecal samples from CRC patients with and without AL. Mice were then subjected to colonic surgery using a colonic anastomosis model. Six days after surgery, anastomotic healing was assessed macroscopically and microscopically. The gut barrier function was also assessed. The gut microbiota composition was compared between the groups colonized with samples from patients with and without AL to detect potential differences.

**Result(s):**

Mice colonized by FMT with the microbiota of donors with AL displayed poor anastomotic healing macroscopically, and a weaker wound microscopically. These same mice displayed a weaker gut barrier, as objectified by higher bacterial translocation to the spleen. The anastomoses of mice receiving the microbiota of AL donors displayed lower concentrations of collagen and fibronectin and higher inflammatory cytokines and collagenolytic enzymes, indicating poor extracellular matrix formation and collagen degradation locally.The beta-diversity of the gut microbiota was significantly different between mice receiving the microbiota of donors with and without AL, and several bacterial species were differentially abundant between the two groups.

**Conclusion(s):**

The preoperative gut microbiota in CRC patients who experience anastomotic leak after surgery induces poor anastomotic healing in mice and a weaker gut barrier after colonic surgery. Several bacterial species were found to be associated with the healing process.

**Please acknowledge all funding agencies by checking the applicable boxes below:**

CIHR, Other

**Please indicate your source of funding;:**

NSERC, FRQS, New Frontiers in Research, Montreal Cancer Institute.

**Disclosure of Interest:**

None Declared

